# Community Health Worker Evaluation of Implementing an mHealth Application to Support Maternal Health Care in Rural India

**DOI:** 10.3389/fgwh.2021.645690

**Published:** 2021-09-01

**Authors:** Umesh Charanthimath, Geetanjali Katageri, Mai-Lei Woo Kinshella, Ashalata Mallapur, Shivaprasad Goudar, Umesh Ramadurg, Marianne Vidler, Sumedha Sharma, Richard Derman, Laura A. Magee, Peter von Dadelszen, Mrutyunjaya Bellad, Beth A. Payne

**Affiliations:** ^1^Department of Obstetrics and Gynecology, KLE Academy of Higher Education & Research J.N. Medical College, Belagavi, India; ^2^Department of Obstetrics and Gynecology, S Nijalingappa Medical College, Bagalkot, India; ^3^Department of Obstetrics and Gynecology, University of British Columbia, Vancouver, BC, Canada; ^4^Department of Obstetrics and Gynecology, Thomas Jefferson University, Philadelphia, PA, United States; ^5^Department of Obstetrics and Gynecology, King's College London, London, United Kingdom

**Keywords:** PIERS on the Move, mHealth, community health workers, accredited social health activists, auxiliary nurse midwives, India

## Abstract

**Introduction:** PIERS on the Move (POM) is a mobile health (mHealth) application developed for a smartphone to support community health workers (CHWs) for identification and management of women at risk of adverse outcomes from pre-eclampsia. POM was implemented as an addition to routine antenatal care by accredited social health activists (ASHAs) and auxiliary nurse midwives (ANMs) during the community level intervention for pre-eclampsia (CLIP) Trial in Karnataka state, India (NCT01911494). The objective of this study was to evaluate the experiences of CHWs of using POM in rural India and their perceptions of acceptability and feasibility of this mHealth intervention.

**Methods:** A posttrial mixed-methods evaluation was designed to measure CHW knowledge and self-efficacy regarding the care of women with pre-eclampsia and perceptions of CHWs on the ease of use and usefulness of POM. A structured survey with open-ended questions was conducted between October and November 2017. The median values on a 5-point Likert scale for knowledge and self-efficacy questions were compared between trial arms by Mann–Whitney U test (*p* < 0.05 significant). Qualitative analysis was undertaken on *NVivo* 12 (QSR International, Melbourne, Australia).

**Results:** A total of 48 ASHAs and ANMs were interviewed, including 24 who used POM (intervention arm) and 24 who did not (control arm). Self-reported knowledge and self-efficacy for the care of women with pre-eclampsia did not differ between groups. The qualitative analysis highlighted that health workers who used POM reported improved interactions with women and families in their communities. POM strengthened the role of ASHA as a CHW beyond a “link-worker” accompanying women to health services. With training, the mHealth application was easy to use even for CHWs who did not have much experience with smartphones.

**Conclusions:** Community health workers found the POM app easy to use, useful, and well-received by women and their families. POM did not improve care through increased knowledge but built capacity by increasing the recognition of the ASHA and ANM as critical members of the continuum of antenatal healthcare within their communities. These findings support the important role that mHealth technologies can play in strengthening health systems to reach rural, remote, and marginalized populations to reduce disparities in health.

## Introduction

There is increasing recognition of the important role that digital health technologies can play in strengthening health systems to increase access for rural, remote, and marginalized populations (1). In May 2018, the World Health Assembly Resolution on Digital Health (WHA/71 A71) acknowledged the potential for digital technologies to support health promotion and disease prevention activities and also improve the accessibility, quality, and affordability of health services (2). Mobile devices are widely used in many settings, including in low- and middle-income countries, where they offer unique opportunities for service delivery at the point of care (3, 4). Mobile technologies for health, mHealth, have the potential to fill gaps in care that result from geographic inaccessibility, low demand for services, delayed provision of care, low adherence to clinical guidelines and protocols, and financial costs for families to seek care that undermine health system performance (1).

However, underlying the optimism of mHealth solutions is an awareness that digital health innovations are not sufficient on their own (5, 6). Digital health innovations need to complement and enhance existing health systems, be appropriate for local contexts, acknowledge the involvement of the health workers who utilize the devices and evaluate implementation factors (4, 5). The WHO guidelines on digital health interventions emphasize the importance of providing supportive environments for health workers to adapt to digital platforms and to be able to use the technology easily (5). Health worker decision support tools, defined by the WHO as “digital job aids that combine the health information of an individual with the knowledge and clinical protocols of a health worker to assist health workers in making diagnosis and treatment decisions” (5), are one area to explore issues of acceptability and feasibility of mHealth innovations.

Conducted in India from February 2014–October 2016, the “Community Level Intervention for Pre-eclampsia (CLIP)” Trial (NCT01911494) sought to reduce maternal, perinatal, and neonatal mortality and morbidity through improving community awareness and CHW capacity to identify pregnant women at risk of pre-eclampsia-related complications during home visits (7). Pre-eclampsia is a pregnancy condition characterized by the new development of hypertension after 20 weeks of gestation alongside signs of maternal organ damage (8), and it is the second leading cause of maternal deaths globally (9). One in ten pregnancies (10.3%) is complicated by hypertension in northwest Karnataka, India, including 3.8% that meets the criteria for pre-eclampsia (10).

The CHW trained to provide the CLIP intervention included accredited Social Health Activists (ASHA) and Auxiliary Nurse Midwives (ANM). As part of the CLIP intervention in India, the CHW training sought to (1) increase their awareness and understanding of pre-eclampsia as a pregnancy complication, (2) make them capable of recording blood pressure (BP) and proteinuria, and (3) make them capable of entering the data on the POM mHealth application and following the advice generated on the application. The POM is a health worker decision support tool that guided the measurement of BP and proteinuria and used this information to provide support regarding decisions to administer drugs to pregnant women or refer them for follow-up care, when the risk of adverse outcomes related to pre-eclampsia was indicated (11). In addition, the ANMs were trained to administer intramuscular magnesium sulfate and oral methyldopa when required to do so. This training was carried out *via* multiple interactions both in groups and one-on-one. Monitoring of activities and refresher trainings were conducted throughout the trial, and any errors or deficiencies were addressed promptly. Contact numbers of the local PHC team and the study personnel were available with the CHWs if support was needed at any time.

To enhance beneficiary awareness and acceptability of the interventions, community engagement sessions were carried out at a minimum frequency of one time in 3 months in each village. Here, pregnant women and stakeholders were advised about birth preparedness and complication readiness and were also specifically educated about the symptoms and signs of the hypertensive disorders of pregnancy, the trial activities in their communities, and the benefits of acceptance and compliance with the trial interventions. These sessions were led by the Medical Officers of the PHC, the staff nurses, and ANMs.

In the control clusters, only data collection activities were added as part of the trial. CHWs were not given any additional training with regard to the intervention activities of the CLIP trial. No community-based activities were undertaken.

The objective of this study, with interviews carried out in October and November 2017, was to evaluate the impact of using the POM app on the knowledge and self-efficacy related to caring for pregnant women, and their perceptions of its usefulness, ease of use, and community reception of ASHAs and ANMs. As part of this, we evaluated the perception of acceptability and feasibility of the current design of the tool by ASHA and ANM and documented recommendations for application improvements. Control cluster CHWs were invited to participate in this exercise to enable a comparison between knowledge and self-efficacy related to caring for pregnant women in those not exposed to the POM application.

## Methodology

### Details on the Intervention Being Studied

PIERS on the Move is a mHealth application that integrates the miniPIERS clinical risk prediction model to calculate the maternal risk of developing a complication of pre-eclampsia (12). POM was developed to support CHWs capacity to identify women at risk of adverse outcomes from pre-eclampsia and guide management, including treatment with oral antihypertensive medication, magnesium sulfate intramuscular injection (MgSO4), and recommendation for transport to the hospital within 4 h or 24 h depending on the calculated risk. As a part of the CLIP trial, CHWs in six intervention clusters in rural India used POM during their home visits to pregnant women as an addition to routine antenatal care.

### Healthcare Worker Evaluation Participants

This is a mixed-methods evaluation study involving healthcare workers who participated in the CLIP India Trial that aims to understand the perspectives of the health workers on community-level pregnancy care and experiences using the POM app. ASHAs are female CHWs who connect rural communities with public health systems (13). Launched in 2005 by the Government of India, ASHAs are recruited from the village they work in, promote good health practices, especially around maternal and child health, and also facilitate access to healthcare services (13). ANMs supervise ASHAs and work at the local health subcenter facilities in the villages. A total of 146 health workers were trained to provide the CLIP intervention; this included 102 ASHAs, seven Anganwadi Workers, 21 ANMS, and 18 staff nurses. There were six intervention clusters and six control clusters. We drew a purposive sample of two ASHAs and two ANMs from each cluster. The ASHAs and ANMs from the intervention clusters were graded according to their compliance with study procedures (e.g., ability to reach women for study visits every 4 weeks) in the CLIP trial to ensure representation of one CHW who performed well and one who performed poorly from each cluster. The ASHAs and ANMs who were chosen in this manner were approached and consented to participation in this evaluation, there being no refusals.

The interviewers who conducted this evaluation were medical doctors who were attached to the research units in India and who were aware of the CLIP study but not a part of it. They had not interacted with these particular CHWs and did not have any administrative authority in the course of their work. For this evaluation, the interviewers were briefed about the trial activities and trainings and the objectives of these interviews. The interviews were carried out one-on-one, with no monetary or other incentives to the interviewers. At the end of the interview, the interviewers went through the completed data form with the CHW and made sure that the options chosen for close-ended questions were indeed what the participant wanted to document.

### Data Collection and Analysis

Data collection with a structured survey including open-ended questions was conducted between October and November 2017 after the CLIP Trial. The questionnaire was translated into the local language, Kannada, and explained to the participants before they answered it. Knowledge and self-efficacy responses were summarized for each question as median [interquartile range] value per group on a 5-point Likert scale. Control and intervention cluster health worker responses were compared, and a *p*-value was estimated using Mann–Whitney *U* test (*p* <0.05 significant).

Qualitative analysis was conducted following grounded theory, which inductively explores themes that emerge from participant open-text responses (14, 15). Four open-text questions were included in the surveys to ASHAs and ANMs:

1. What aspects of work did you find most enjoyable?
2. What problems did you encounter with the POM app?
3. What features of the POM app did you find most useful?
4. Any changes recommended for the POM app?

The first question (question 1 shown above) was asked to ASHAs and ANMs in both the intervention and control clusters, while the remaining three questions (questions 2–4) were asked to health workers in intervention clusters only since POM was part of the clinical intervention and not used in the control clusters. A preliminary analysis was conducted by the primary qualitative coder (MWK) to develop codes for full analysis on NVivo 12 (QSR International, Melbourne, Australia). Findings at each stage from preliminary to full analysis were discussed, clarified, and refined collaboratively between a team of social scientists, clinicians, and epidemiologists in India and Canada who oversaw the CLIP India Trial (UC, GK, AM, SG, UR, MV, SS, LM, PvD, MB, and BP). Lastly, emergent themes were considered in the context of quantitative results to triangulate key findings from the mixed-methods study.

## Results

### Participant Characteristics

A total of 48 ASHAs and ANMs were interviewed in the study, with 24 each in control and intervention clusters. Median age and years of work experience were similar between groups, around 38 years old and almost 10 years of experience in their current positions, respectively (as shown in Table 1).

**Table 1:** Characteristics of the respondents.

	**Control cluster**	**Intervention cluster**
Total number *ASHA* *ANM*	24 *12 (50%)* *12 (50%)*	24 *12(50%)* *12 (50%)*
Age (yrs)	38.0 [31.5−50.0]	38.5 [32.5–46.0]
Worked as ASHA/ANM (yrs)	9.8 [8.2–20.3]	9.6 [7.7–18.1]

### Health Worker Knowledge and Self-Efficacy in Pregnancy Care

All healthcare workers, whether in the intervention or control groups, self-reported high levels of confidence in their knowledge and skill to provide care to pregnant women. The majority of the women reported scores of four or five, indicating agree or strongly agree to knowledge and self-efficacy statements around pregnancy care, identification of maternal complications, and basic clinical care (as shown in Table 2). Differences were not significant between intervention and control clusters, with the exception of two statements around the perceptions of client trust of healthcare workers in their medical knowledge. ANMs agreed or strongly agreed that they could confidently administer injectable vaccines to mothers and intramuscular injections to pregnant women at risk of seizure without a significant difference between intervention and control arms. Though scores ranged between four and five for both control and intervention clusters, ASHAs and ANMs in the control clusters tended to agree stronger than those in intervention clusters with the two statements, “If a woman needs medical attention, she takes my counseling seriously” and “Families trust the health care advise I provide to pregnant women in their community” (*p* < 0.05).

**Table 2:** Knowledge and self-efficacy responses.

	**Control cluster (*N* = 24)**	**Intervention cluster (*N* = 24)**	***p***
I can confidently provide care to pregnant women	5 [5–5]	5 [4.5–5]	0.272
I can confidently provide basic knowledge about pregnancy to the pregnant women I care for	5 [5–5]	5 [5–5]	0.225
I can confidently recognize pregnancy related complications	5 [4.5–5]	5 [4–5]	0.355
I can confidently recognize danger signs / symptoms related to pre-eclampsia and eclampsia	5 [4–5]	5 [5–5]	0.140
I am confident that I can recognize signs and symptoms of labor	5 [5–5]	5 [5–5]	0.670
I am confident that I can accurately check blood pressure	5 [5–5]	5 [5–5]	0.066
Women/families in my community seek my advice on pregnancy and maternal health	5 [5–5]	5 [4–5]	0.126
I feel comfortable accompanying a pregnant woman who needs medical attention to the hospital	5 [5–5]	5 [4.5–5]	0.746
If a woman needs medical attention, she takes my counseling seriously	5 [4–5]	4 [4–5]	0.018
Families trust the health care advise I provide to pregnant women in their community	5 [4–5]	4 [4–5]	0.045
The doctors and nurses in my community view my role as a community health worker as important	5 [5–5]	5 [4.5–5]	0.447

Healthcare workers from intervention clusters reflected on positive professional interactions with clients and their families as what they most enjoyed about their work. For example, in the quotes below, healthcare workers from intervention clusters spontaneously shared how they appreciated that their health advice was valued by pregnant women and their community members, which was not discussed in responses by participants from control clusters.

“*When an expected woman delivers a healthy baby and when she listen to my advice seriously.” 41-year-old ASHA from Kanagala*

“*By taking care of mother and baby, by helping a poor pregnant woman and by responding to all their problems, I feel very happy. As I helped them, they would also appreciate me and I feel happy.” 37-year-old ASHA from Amingad*

“*When I work in my community…when a woman seeks treatment before some mishap occurs by identifying danger signs based on my health education, I feel very happy. I feel that the health education I gave has reached them.” 28-year-old ANM from Pattadakall*

### Health Worker Experiences of Using POM

#### Training

Relatively few health workers owned a smartphone prior to the CLIP trial (*n* = 4, 16.7%), but respondents shared that they felt the training to use the mHealth application and skills required to care for pregnant women were effective (as shown in Table 3). Health workers largely found the POM app easy to use, especially after the training was conducted in their local language of Kannada, and training both the ASHAs, as frontline users of the POM app and their supervisors and ANMs, who staffed the local health facility in the village.

**Table 3:** Summary of survey responses related to experience with the POM app in the CLIP trial.

	**Intervention cluster respondents (*N* = 24)**
Currently owns a smart phone (*n*(%))	18 (75.0%)
Owned smart phone prior to CLIP trial (*n*(%))	4 (16.7%)
Features regularly used on mobile phoneVoice callsText/ SMS messagingGamesOther^*^	23 (95.8%) 16 (66.7%) 7 (29.2%) 13 (54.2%)
The POM app was easy to use	4 [4–5]
I was effectively trained to use the POM mobile application in the CLIP Trial	5 [5–5]
The training I received from the CLIP team provided the skills I needed to care for pregnant women in the CLIP Trial	5 [5–5]
I could rely on the support of the CLIP team to solve problems encountered when using the POM app	5 [4.5–5]
The pregnant women I cared for always reacted positively to my use of the POM app	5 [5–5]
I felt good about using the POM app	5 [5–5]
I felt confident caring for pregnant women in the CLIP trial	5 [4.5–5]
I often had trouble completing study visits using the POM app	4 [2.5–4]
The RA/ARA I work with during the CLIP trial actively supported my use of the POM app	5 [4–5]
The pictograms in the application were useful in helping me counsel women	5 [5–5]
The pictograms adequately conveyed the symptoms they were supposed to represent	5 [5–5]
The refresher trainings helped me overcome obstacles	4.5 [4–5]
The POM app helped me advise women about pregnancy complications	5 [5–5]
Women took my counseling more seriously because of the POM app	5 [4–5]
I would recommend use of the POM app to other health workers	5 [4–5]
If offered, I would choose to continue using the POM app to care for pregnant women	5 [5–5]

* *6=calculator; 9=calendar; 3=maternal and child health tracking app as part of government initiative; 5=movies or music*.

“*If I entered wrong data in the app it was difficult for me to correct it, but later they trained me and I got information about it.” 37-year-old ASHA from Amingad*

“*Initially I was not able to use smart phones. But they explained (trained) me very well in it's use.” 60-year-old ANM from Amingad*

“*They explained me in my language so that I should not face any difficulties. Hence, it was easy for me to work.” 51-year-old ANM from Amingad*

#### Useful Knowledge and Skills

Health workers shared that they felt confident using the POM app, helping them identify and advise women about pregnancy complications and guiding the management of conditions (as shown in Table 3). While ASHAs utilized the POM app during home visits, ANMs working at the village subcenter health post shared their appreciation for clinical indicators. ASHAs and ANMs described the usefulness of clinical indicators, risk predictions, and referral recommendations to strengthen their health counseling and care for pregnant women in their communities.

“*The POM app used to show signs and symptoms of high blood pressure that aspect helped me a lot” 43-year-old ASHA from Hosur*

“*POM app was very helpful to me for taking care of pregnant woman… Entering the pregnant woman's LMP, EDD, Weight, Height, BP. overall all information. [made] it easy to detect whether she is in danger.” 60-year-old ANM from Amingad*

“*If the blood pressure was high, POM app used to guide us, how to manage further.” 41-year-old ASHA from Kanagala*

“*Entering BP value, urine [protein value] and popping up of ambulance and lady's pictures were very helpful.” 27-year-old ASHA from Sutagundar*

#### Positive Reception From Pregnant Women

Participants strongly agreed that the POM app was well received by the pregnant women they cared for, that the pictures made it easier to explain maternal health conditions, and that the POM app helped them advise women about pregnancy complications (as shown in Table 3).

“*In the community, the POM app was very useful because it had the pictures of all the symptoms of high blood pressure… By seeing the pictures of the symptoms, it was easy for them to understand what problems they will have when the BP is high. This was very helpful for them.” 37-year-old ASHA from Amingad*

“*Explaining about eclampsia via charts was helpful. Otherwise, people could not have understood.” 52-year-old ANM from Sutagundar*

#### Recommendation for Scaling Up POM

Health workers in the study supported scaling up the use of the POM health to other health workers and in their own use after the intervention concluded (as shown in Table 3).

“*POM should have been given to all ASHA's and it should be permanent work (wants to continue the use of it)” 27-year-old ASHA from Sutagundar*

“*This service should be available everywhere.” 27-year-old ASHA from Sutagundar*

“*Giving the POM to all ASHAs and ANMs after more training and asking them all to use it for all pregnant women will be helpful in preventing complications in pregnant women. We will be able to get a healthy mother and baby by doing this.” 60-year-old ANM from Amingad*

However, it is of note that a number of health workers shared that they often had trouble completing study visits (as shown in Table 3).

### Challenges Using POM

Challenges using the POM mHealth app included both device and app issues (as shown in Table 4). Key application-related challenges included the complexity of editing data (*n* = 11, 45.8%) and the length of the application with many app fields to fill in (*n* = 10, 41.7%). A key device issue that the health workers reported was that the phone date often reset, which was shared by half of the respondents (*n* = 12, 50.0%). This issue was discovered during the pilot trial, and consequently, a different brand of smartphone was sourced for the main trial. The second leading device challenge was that the battery life of the phone was insufficient (*n* = 6, 25.0%). Respondents shared that these two device issues were potentially linked.

**Table 4:** Issues reported with POM app during the trial.

	**Respondents reporting issue (*N* = 24)**
a. Device issues–battery life of phone insufficient	6 (25.0%)
b. Device issues–screen was too small	5 (20.8%)
c. Device issues–the phone date often reset	12 (50.0%)
d. Device issues–the app ran slowly	3 (12.5%)
e. App issues–updates/changes to the app occurred too frequently	4 (16.7%)
f. App issues–too many fields to fill in	10 (41.7%)
g. App issues–correcting mistakes and editing data was too complex	11 (45.8%)
h. App issues–the language was inaccurate and hard to understand	2 (8.3%)
i. App issues–hard to navigate through the screens to complete the tasks	4 (16.7%)
j. App issues–previous visit data could not always be found	3 (12.5%)

“*Dates shown in POM app used to change [to the wrong date] frequently that caused me inconvenience.” 43-year-old ASHA from Amingad*

“*The battery used to get discharged. Needed to charge again and again. Had to keep correcting the date.” 27-year-old ASHA from Sutagundar*

Additional issues that were reported included trouble with synchronization, a request to add more local languages, the size of the phone being small, and the difficulty understanding how to ID women at first. Challenges in correcting dates, entering information, and overall using the app were frequently resolved with training, refreshers, and support from ANM for ASHAs using POM in the communities.

“*Initially I had great difficulty in correcting the date on the mobile. This was later corrected by my higher ups.” 37-year-old ASHA from Amingad*

*POM app opening code entry was not correct. Some ASHA's were not able to enter in POM app; they used to do so with the help of the ANMs” 27-year-old ANM from Pattadakall*

### Health Worker Recommendations to Improve POM

Many ASHAs and ANMs (*n* = 10, 41.7%) shared that no changes were needed. The leading change to the POM app to make it more useful to health workers was adding anemia indicators (*n* = 9, 37.5%).

“*Can add HB testing to make POM app more useful.” 43-year-old ASHA from Amingad*

“*If you can add Hemoglobin [to the] application, it will be useful for quality ANC care” 32-year-old ANM from Sangolli*

Speeding up the application, more local languages, and audio aids in addition to the visual aids and incorporating mobile number and address for follow-up were also suggested by health workers to improve the system.

## Discussion

### Main Findings

The findings demonstrate that the use of the POM mHealth application was feasible with appropriate training and support systems for rural health workers in India that previously did not have much experience with smartphones. There were technical challenges with the battery life of the device, entering and editing data, and data synchronization, but many issues were resolved with training in the local language and conducted with both ASHAs and their supervisors, ANMs. Overall, rural health workers in our study found the POM app easy to use and useful in their work with pregnant mothers in their communities and well-received by women and their families. Though self-reported knowledge and self-efficacy did not differ between intervention and control clusters, health workers who used POM reported that pictograms describing the symptoms were especially helpful to explain maternal conditions. The app was an effective platform for distilling obstetric knowledge of a panel of experts into an accessible, pictogram-based, and algorithm-driven format that was readily usable by lay cadres of health workers in community settings.

### What Is Already Known on This Topic

A national longitudinal study in India found a positive impact of ASHAs on utilization of maternity services, including a 28% increase in facility births and a 17% increase in at least one antenatal visit associated with ASHA services between 2004 and 2012 (16). However, while ASHAs are valued for their contribution toward maternal health education, studies in rural Manipur (17) and Karnataka state (18) find that ASHAs are largely understood as a link-worker that connects community members with health services but not as a CHW or a social activist as they are intended. The study in Karnataka, the same state as this study, found that ASHAs were predominately involved in-home visits, antenatal counseling, delivery escort services, breastfeeding, and immunization advice, but the performance was low for contraceptive use, obstetric danger sign assessment, and newborn care (18). Poor identification of obstetric complications was also found in another study in rural Karnataka where researchers found that less than 8% of ASHAs were aware of the pregnancy, labor, and postpartum danger signs (19). Regarding pre-eclampsia, in particular, a qualitative study found that ASHAs and ANMs in rural Karnataka had limited knowledge of the condition and treatment (20). Additionally, poor reception and quality of care at primary health centers that ASHAs referred to had negative consequences in community trust in the credibility of their health knowledge and skills (17).

Poor fulfillment of CHW and social activist roles may be related to health system constraints, poor capacity to provide support, oversight,and inadequate training (21). While ASHAs are ideally literate and have completed secondary school (standard 10), standards are flexible to the availability of women interested in the position in each community (13). Training to ASHAs is provided in a series of modules over the course of a year (13, 16). However, a study in Maharashtra, India, found that nearly half of the ASHAs in their study did not have any secondary education, and knowledge gaps existed even after the training modules (22). The authors recommended that a platform for continual capacity building and reinforcement of health information would be helpful (22).

An exploration of the potential for mHealth for cardiovascular disease management in Kerala, India, found that a mHealth platform could function as an educational tool to improve health education counseling, help improve ASHA job efficacy in reaching rural and marginalized populations, and improve the use of healthcare services through appointment and treatment reminders (23). Health workers in the study highlighted the need for mHealth designs to integrate activities with local health systems and complement existing clinical practices instead of developing a parallel system (23). Observations (24) and randomized controlled trials (25, 26) have shown that adoption of mHealth technologies by ASHAs can positively impact coverage and quality of maternal and child health services by increasing the number of home visits, and provisioning of supportive supervision and greater job confidence among frontline health workers. Supportive supervision, training, ongoing technological support, health system preparedness, and some individual characteristics such as literacy and age were identified as factors that influence mHealth adoption among ASHAs (24, 26).

More broadly, a review for the WHO guidelines on digital health found that the health worker acceptability was supported by expanding their range of tasks, increased speed and efficiency of their work, the ability to connect with different people and health service levels to coordinate care delivery, and increased respect received from health professionals and from the community (5). Acceptability of mHealth tools was hindered when it increased health worker workload, especially if it is not appreciated or used by their supervisors, and when they did not perceive the intervention as useful for their work in the communities (5). Health workers who struggle to use digital health technologies are likely to have negative perceptions about their usefulness, and the acceptability of digital health interventions may be shaped by pre-existing digital literacy of the health workers. Feasibility barriers include infrastructural challenges such as poor network connectivity, access to electricity for charging, and challenges in the program design or device, such as not in the local language, small screens, devices being ill-suited for note-taking, and SMS character limitations (5). Adequate training and familiarity may help overcome some of these difficulties (5).

### What This Study Adds

Building on previous research studies with ASHAs in Karnataka and other areas in India that identified a gap in the recognition of maternal and neonatal complications (18–20, 22), this study demonstrated that the mHealth platform guided practice in the presence of obstetric danger signs. The use of clinical indicators of POM strengthened the referral process and communication of maternal risk across different service levels and complemented existing health systems and clinical practices instead of creating parallel systems. With the expansion of their knowledge and skills into clinical indicators of maternal danger signs, the POM app may have strengthened the role of ASHAs as CHWs beyond common perceptions of their role as link-workers that escort women to health services (17, 18). However, it is of note that intervention cluster ASHA and ANM reported slightly lower scores than their counterparts in control clusters on community trust in their health counseling. Though these may not be meaningfully different, the responses may indicate heightened awareness of their role as advice-givers and counselors providing healthcare information through the app. Additionally, regular feedback during monthly monitoring reports may be associated with a higher degree of awareness of their performance among health workers in the intervention arm compared with those in the control arm. Furthermore, changing the role of ASHA from link-worker to care provider may have been initially met with some resistance in some areas.

The study adds to the evidence that expanding the range of tasks, increased respect received from the community, and perceptions that the mHealth tool was easy to use and was useful for their work of caring for pregnant women supported health worker acceptability. Feasibility challenges such as battery issues, data entry, editing, synchronization, and long length of forms to complete were highlighted, but they did not seem to prevent health workers from recommending scale-up of the tool or request the addition of anemia indicators to the tool.

While others have suggested that health workers with low preexisting digital literacy may have difficulties transitioning to mHealth technologies and be associated with lower acceptability and perceptions of its usefulness (5), our study demonstrated the ability to introduce a mHealth app to a population of health workers with low previous experience with smartphones. One potential reason may be the use of pictograms to illustrate symptoms, which were reported to be well-received by health workers and community members alike. Though presented on a digital platform, the use of pictograms may have been well suited to local rural contexts where there may be high rates of overall illiteracy, especially among women. A second reason may be the provision of training in the local language and training both community outreach workers (ASHAs) and the staff at the village level health subcenters who supervised them (ANMs). A previous study has suggested that the use and value of a new technological system of supervisors support acceptability by workers through conformity to social norms (27). Additionally, the knowledge and value of the supervisors on the new system were a support system for health workers when challenges occurred. In addition to ANM support, the ASHAs in our study were also supported by the research project team when there were technological difficulties. A third reason may be the increasing use of mobile technologies in the communities outside of the intervention that led to more familiarity with digital systems.

### Strengths and Limitations of the Study

Strengths of the study were interviewing both ASHAs and ANMs for their perspectives on the mHealth intervention, a mixed-method design that incorporated both quantitative and qualitative methodologies, and strong collaboration between Indian and Canadian researchers in the CLIP Trial and in the analysis and interpretation of health worker evaluation results. Additionally, physicians outside of the CLIP study facilitated data collection to reduce the potential for bias.

The use of a Likert scale was a limitation of the study. While Likert scales allow for degrees of opinion rather than only a yes or no response, research study has shown that there can be cultural differences in response to Likert scales, as some cultures were more biased to reporting positive emotions (28). Consequently, there may be a possibility that the overall positive responses in our quantitative research study may be related to cultural tendencies to report positivity or social desirability to respond positively to the research staff. Based on the positive knowledge scores in both control and intervention clusters, we suspect that some reporting bias remained due to the power imbalance between CHW and physician data collectors.

There may also be a positive bias to the findings, particularly the recommendation to scale-up the use of the app, due to its integration within an intervention study and the incentives health workers received from the research project. ASHAs are considered honorary volunteers who receive some performance-based monetary incentives upon escorting women to health services (16) but may receive more regular stipends in an intervention study. A review found that studies with ASHAs within special interventions had largely positive results in contrast to more negative findings in studies with routine ASHA work, suggesting the importance of health system constraints (21). A limitation of this study was that it focused on perceptions of ASHAs and their immediate supervisors but did not examine beyond the community level or fully investigate health system constraints. A number of health workers shared that they often had trouble completing study visits, suggesting that there may have been larger constraints at play that could be further explored in future studies.

## Conclusions

The health worker evaluation into perceptions and experiences of ASHA of using the POM app acknowledges the involvement of health workers who utilize the mHealth technology and seeks to understand the challenges and enablers to their use. Though many of the health workers involved in the CLIP India Trial did not previously own a smartphone, they reported that the POM app was both acceptable and feasible for strengthening their care of pregnant women in the communities they work in. Utilization of the POM app to support maternal healthcare at the community level built capacity by increasing the recognition of the ASHA and ANM as critical members of the continuum of antenatal health care within their communities. These findings support the important role digital health technologies can play in strengthening health systems to reach rural, remote, and marginalized populations to reduce disparities in health.

## Data Availability Statement

The raw data supporting the conclusions of this article will be made available by the authors, without undue reservation.

## Ethics Statement

The studies involving human participants were reviewed and approved by KLE University (MDC/IECHSR/2011-12/A-4; ICMR 5/7/859/12-RHN) and the University of British Columbia (UBC, H12-03497). The patients/participants provided their written informed consent to participate in this study.

## Author Contributions

PvD, MB, SG, RD, BP, MV, and LM conceptualized the trial and designed the components of the intervention. BP, GK, UC, UR, AM, MB, and SG designed the health worker evaluation. GK, UC, AM, SG, and UR implemented the health worker evaluation and were responsible for data collection, transfer, and data cleaning. MWK and MV performed the analyses. MWK and BP wrote the first draft of the manuscript. All the authors provided feedback and review of the manuscript.

## Conflict of Interest

The authors declare that the research was conducted in the absence of any commercial or financial relationships that could be construed as a potential conflict of interest.

## Publisher's Note

All claims expressed in this article are solely those of the authors and do not necessarily represent those of their affiliated organizations, or those of the publisher, the editors and the reviewers. Any product that may be evaluated in this article, or claim that may be made by its manufacturer, is not guaranteed or endorsed by the publisher.
