# Supplementary material for: Community Health Worker Evaluation of Implementing an mHealth Application to Support Maternal Health Care in Rural India
Source: Front Glob Womens Health. 2021 Sep 1;2:645690. doi: 10.3389/fgwh.2021.645690 (PMC8593958; doi:10.3389/fgwh.2021.645690)
Supplement: Supplementary file 1 [file Table_1.DOCX]

**Table S1 CLIP Trials Working Group for appearance on PubMed**

| **First and middle names** | **Last names** |
| --- | --- |
| Shashidhar G | Bannale |
| Keval S | Chougala |
| Vaibhav B | Dhamanekar |
| Anjali M | Joshi |
| Namdev A | Kamble |
| Gudadayya S | Kengapur |
| Uday S | Kudachi |
| Sphoorthi S | Mastiholi |
| Geetanjali I | Mungarwadi |
| Esperança | Sevene |
| Khátia | Munguambe |
| Charfudin | Sacoor |
| Eusébio | Macete |
| Helena | Boene |
| Felizarda | Amose |
| Orvalho | Augusto |
| Cassimo | Bique |
| Ana Ilda | Biz |
| Rogério | Chiaú |
| Silvestre | Cutana |
| Paulo | Filimone |
| Emília | Gonçálves |
| Marta | Macamo |
| Salésio | Macuacua |
| Sónia | Maculuve |
| Ernesto | Mandlate |
| Analisa | Matavele |
| Sibone | Mocumbi |
| Dulce | Mulungo |
| Zefanias | Nhamirre |
| Ariel | Nhancolo |
| Cláudio | Nkumbula |
| Vivalde | Nobela |
| Rosa | Pires |
| Corsino | Tchavana |
| Anifa | Vala |
| Faustino | Vilanculo |
| Rahat N | Qureshi |
| Sana | Sheikh |
| Zahra | Hoodbhoy |
| Imran | Ahmed |
| Amjad | Hussain |
| Javed | Memon |
| Farrukh | Raza |
| Olalekan O | Adetoro |
| John O | Sotunsa |
| Sharla K | Drebit |
| Chirag | Kariya |
| Mansun | Lui |
| Diane | Sawchuck |
| Ugochi V | Ukah |
| Mai-Lei Woo | Kinshella |
| Shafik | Dharamsi |
| Guy A | Dumont |
| Tabassum | Firoz |
| Ana Pilar | Betrán |
| Susheela M | Engelbrecht |
| Veronique | Filippi |
| William A | Grobman |
| Marian | Knight |
| Ana | Langer |
| Simon A | Lewin |
| Gwyneth | Lewis |
| Craig | Mitton |
| Nadine | Schuurman |
| James G | Thornton |
| France | Donnay |
| Romano N | Byaruhanga |
| Brian | Darlow |
| Eileen | Hutton |
| Mario | Merialdi |
| Lehana | Thabane |
| Kelly | Pickerill |
